# Three patients with both Hodgkin's lymphoma and Castleman's disease: Clinicopathologic correlations and lack of association with HHV-8

**DOI:** 10.4103/0971-5851.60052

**Published:** 2009

**Authors:** Saadiya Haque, Robert van Kirk

**Affiliations:** *Georgetown University Hospital, 3900 Reservoir Road, Washington, DC 20007*; 1 *Prince Williams Hospital, 8700 Sudley Road, Manassas, VA 20110, USA*

**Keywords:** *Castleman's disease*, *Hodgkin's lymphoma*, *HHV-8*, *plasma cell variant of Castleman's disease*

## Abstract

**Background:**

The relationship between Hodgkin's lymphoma (HL) and plasma cell-type Castleman's disease (PCD) has been well documented. There have been over 20 cases reported in the literature and nearly all of them were either diagnosed concurrently, or were initially diagnosed as PCD and upon review were found to have interfollicular HL. Human herpes virus type 8 (HHV-8) is present in about 40% of cases with PCD. It predisposes patients to a much higher risk of other malignancies, including Kaposi's sarcoma and non-Hodgkin's lymphoma. Cases linked to HHV-8 are associated with a different morphology than cases that are not linked to HHV-8. It has been proposed that patients with both HL and CD will have lymph nodes with HHV-8-negative morphology.

**Materials and Methods:**

We present a series of three cases in a retrospective study where patients had both HL and PCD. Surgical pathology reports, clinical histories, and H and E and various immunohistochemical stains on initial work-up were examined and subsequent immunohistochemical stains for HHV-8 were obtained from the Methodist Hospital.

**Results:**

Patient 1 was diagnosed with PCD and interfollicular HL in the same lymph node. Patient 2 was first diagnosed with classic HL and 2 years later returned with enlarged lymph nodes clinically suspected to be recurrent HL. Histology showed angiofollicular hyperplasia and interfollicular plasmacytosis without Reed-Sternberg cells and a diagnosis of PCD was rendered. Patient 3, a male in his third decade, was diagnosed with nodular sclerosing HL in the thymus, and concurrently PCD in the mediastinal lymph nodes. All three cases had architectural features consistent with an HHV-8-negative morphology. Immunohistochemical stains for HHV-8 were done retrospectively and were negative.

**Conclusion:**

All three of our patients with both HL and CD had HHV-8-negative lymph node morphology and absence of HHV-8 by immunohistochemistry. These patients, therefore, are not at an increased risk for the development of subsequent malignancies, when compared to HHV-8-positive patients. Included in our series is one unique case where the diagnosis of HL preceded CD by 2 years.

## INTRODUCTION

The concept of Castleman's disease (CD) (angiofollicular lymph node hyperplasia, “giant” lymph node hyperplasia, angiomatous lymphoid hamartoma) has been evolving since Castleman first described it in 1956.[1] This disease is now recognized to include a widely variable range of clinical presentations, histological findings, variable disease course, and ancillary study findings. Clinical presentations range from an incidental finding in the course of an unrelated health issue to multiple systemic symptoms and syndromes. The tumor can be localized and solitary or systemic and multicentric. Histologically, tissues may be classified as hyaline vascular type, plasma cell type, or mixed type. The disease course may range from an asymptomatic course to a progressive and fatal outcome.

CD has been associated with multiple viruses. Plasma cell-type CD (PCD) is often associated with human immunodeficiency virus (HIV), especially when multicentric. Recent discoveries have led to a new way of classifying variants of PCD, based on the presence of human herpes virus type 8 (HHV-8). Cases linked to HHV-8 have a different histopathology, clinical course, and likelihood of developing lymphoma[2] when compared to HHV-8-negative cases.

A relationship between Hodgkin's lymphoma (HL) and PCD has been well documented. There have been 29 cases reported in the literature and nearly all of them were either diagnosed concurrently, or were initially diagnosed as PCD and upon review were found to have interfollicular HL.[3–7] To our knowledge, the HHV-8 status of these types of cases has not been reported, with the exception of few case reports.[8] In the following study, we report three cases of patients with both HL and CD and classify them by HHV-8 status based on morphology and immunohistochemical studies.

## MATERIALS AND METHODS

Three patients are included in this series. Cases 1 and 2 were obtained from the Metro Health Medical Center archives. Case 3 was obtained from the Medical College of Wisconsin. Surgical pathology reports and clinical histories were obtained from the hospital information systems.

All cases initially had H and E and various immunohistochemical stains on initial work-up. Immunohistochemical stains for HHV-8 were done by Methodist Hospital (Houston, TX) retrospectively.

## RESULTS

**Figure 1:**
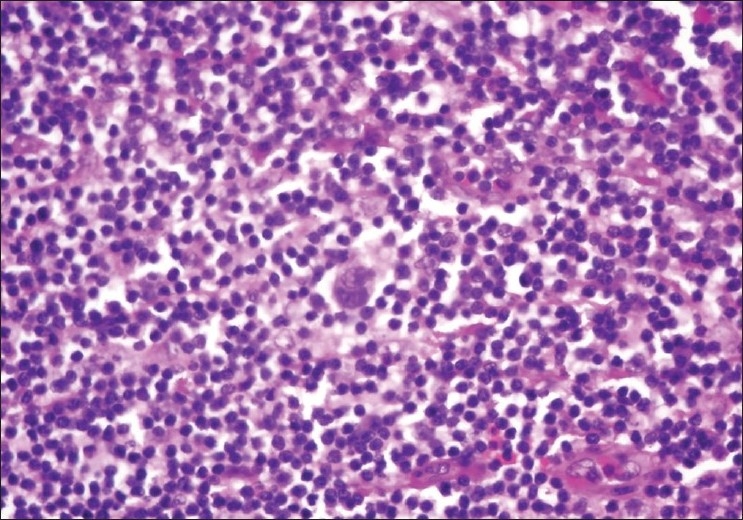
Case 1. Reed-Sternberg cells in the interfollicular area surrounded by numerous plasma cells, H and E stain, ×40

Case 1, a 62-year-old HIV-negative female, was diagnosed with PCD and interfollicular HL concurrently in the same mediastinal lymph node [Figure 1]. The node morphology showed intact follicles and distinct mantle zones.

**Figure 2:**
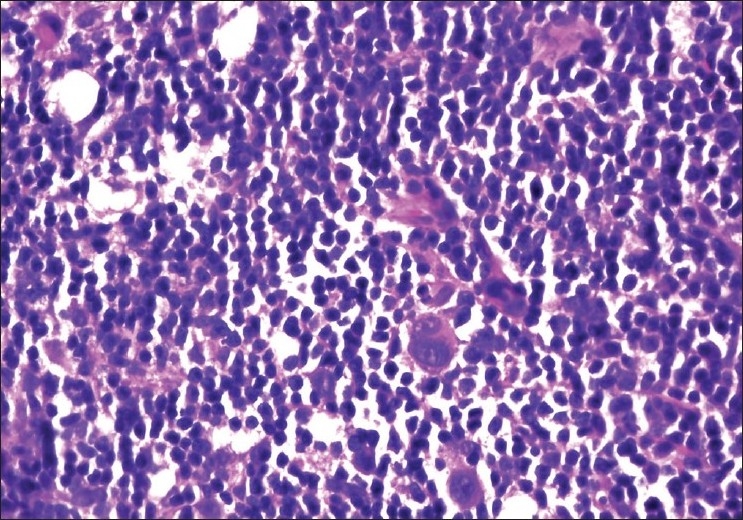
Case 2. Bilobed Reed-Sternberg cells found in an effaced lymph node, H and E stain, ×40

Case 2 was a 67-year-old HIV-negative male who was first diagnosed with mixed cellularity HL in a cervical lymph node [Figure 2]. Following treatment with chemotherapy and 10 months of remission, the patient returned with enlarged axillary lymph nodes clinically suspected to be recurrent HL. Histology showed angiofollicular hyperplasia and interfollicular plasmacytosis without Reed-Sternberg cells and a diagnosis of PCD was rendered. The follicles were intact, without lysis or hemorrhage, and mantle zones were well delineated.

**Figure 3:**
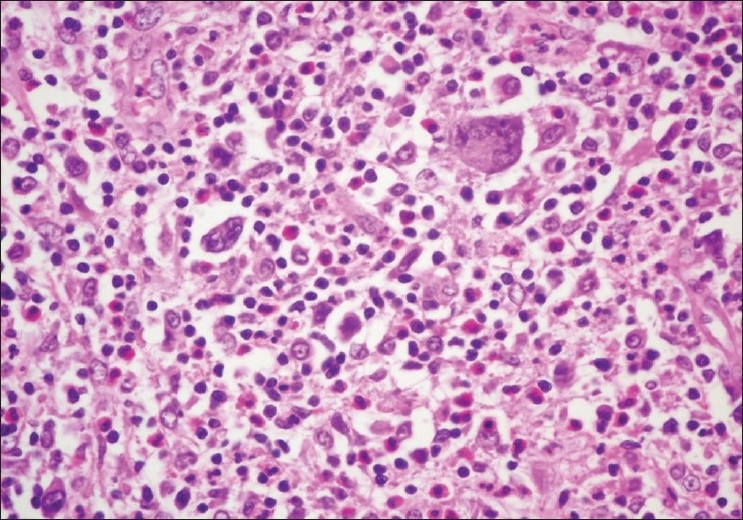
Case 3. Nodular lesions with several Reed-Sternberg cells, H and E stain, ×40

Case 3, a 27-year-old HIV-negative male, was diagnosed with nodular sclerosing HL in the thymus [Figure 3] and concurrently PCD in the mediastinal lymph nodes.

**Figure 4:**
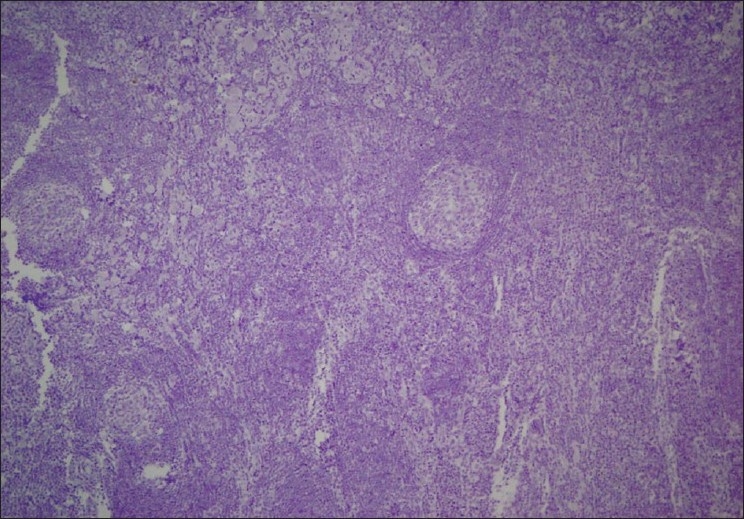
HHV-8 stain was negative for all three patients, HHV stain, ×20

All three cases had architectural features consistent with an HHV-8-negative morphology. Immunohistochemical stains for HHV-8 were negative in all cases [Figure 4].

## DISCUSSION

Our study validated that cases associated with both HL and CD typically demonstrate an HHV-8-negative lymph node morphology.

HHV-8 is present in about 40% of cases with multicentric PCD and a minority of cases with localized PCD.[9] It predisposes patients to a much higher risk of other malignancies, including Kaposi's sarcoma and non-Hodgkin's lymphoma and is associated with a worse prognosis.

HHV-8-positive cases are associated with a different morphology than HHV-8-negative cases.[2] HHV-8-negative cases have enlarged, active, hyperplastic follicular centers with mitoses, macrophages with nuclear debris, and increased follicular dendritic cells. There is usually a thin but distinct mantle zone. HHV-8-positive PCD cases have follicles that show severe hemorrhage and lysis with dissolution of the germinal center. The mantle zone-interfollicular area boundary is obliterated with the presence of indistinct, irregular mantle zones. Additionally, HHV-8-positive cases are more likely to have plasma cell atypia with immunoblasts and plasmablasts present in the mantle zones and more pronounced vascular proliferation in the interfollicular areas. There is also intranodal sinus dilatation which is absent in HHV-8-negative cases.[2] All three of the patients in our series had a morphology consistent with the HHV-8-negative type of PCD.

The etiology of this apparently heterogenous group of diseases currently classified as CD is not well understood. Numerous authors have tried to implicate IL-6 (a cytokine normally produced in the germinal center that serves to induce B-cell differentiation) because it is seen with an increased expression in PCD and most patients respond to treatment with anti IL-6 antibodies.[10–12] This may help explain the HHV-8-associated changes because HHV-8 encodes an IL-6 homolog.[13] In CD with concurrent HHV-8 infection, there is a total increase in the systemic levels of IL-6, which causes the different morphologies seen in HHV-8-positive cases.

It has been suggested that CD should be divided into primary and secondary, with secondary being associated with conditions that result in an IL-6-rich environment.[10–12] This would include HIV, autoimmune disorders, and plasma cell dyscrasias. It has also been shown that Reed-Sternberg cells and mononuclear-variant cells produce IL-6.[11]One report says of 19 cases with both HL and CD, that the diagnosis of Hodgkin's disease was delayed in 16 cases.[3] The Castleman-like changes induced by IL-6 can make it harder for the pathologist to recognize the diagnostic Reed-Sternberg cells, and consequently, the diagnosis of a very treatable disease may then be missed.[3–7] Our series included one case (case 1) of interfollicular HL that was diagnosed concurrently in a mediastinal lymph node. Another case (case 3) presented concurrently with nodular sclerosing HL in the thymus and PCD in a mediastinal node. The most unusual in our series was the case 2 who presented with mixed cellularity HL first, and then 16 months later (10 months postchemotherapy induced remission) returned with PCD. To our knowledge, this is the first case of its kind.

Our three patients each had a different subtype of classical HL (interfollicular, nodular sclerosing, mixed cellularity). This is not unusual because previous reports in patients with both HL and PCD included all types of classical HL as well as nodular lymphocyte-predominant HL.

## CONCLUSION

It has been proposed that patients with both HL and CD will most often have lymph nodes with an HHV-8-negative morphology,[2] but our series is the first report to verify this concept in multiple patients. Our series of three cases showed morphology with HHV-8-negative features and were negative for HHV-8 by immunohistochemical studies. Included is the first reported case of HL preceding the PCD changes.
